# Poster 1023: Dupilumab suppresses Th2 inflammation in adult asthma and atopic dermatitis

**DOI:** 10.1186/1939-4551-7-S1-P13

**Published:** 2014-02-03

**Authors:** Brian Swanson, Jeffrey Ming, Haobo Ren, Lin Wang, Sally Wenzel, Lisa Beck, Thomas Diciccio, Yongtao Li, Pavel Belomestnov, Neil Graham, Gianluca Pirozzi, Jennifer Hamilton

**Affiliations:** 1 Sanofi, Bridgewater, NJ, USA; 2 Regeneron Pharmaceuticals, Inc., Basking Ridge, NJ, USA; 3 University of Pittsburgh, Division of Pulmonary, Allergy, and Critical Care Medicine, Pittsburgh, PA, USA; 4 University of Rochester, School of Medicine & Dentistry, Rochester, NY, USA; 5 Regeneron Pharmaceuticals, Inc., Tarrytown, NY, USA

## Background

Dupilumab (DPL) is a fully human IL-4Rα monoclonal antibody that potently inhibits both IL-4 and IL-13 signaling, drivers of T-helper 2 (Th2) mediated inflammation. In early clinical trials, DPL has recently been evaluated in proof-of-concept studies in asthma and atopic dermatitis (AD).

## Methods

The clinical trial design and results in adult patients were previously reported for asthma (*NEJM* 2013;368:2455) and AD (*JID* 2013;133:S177-S178). In the asthma study, inhaled glucocorticoids and long-acting beta-agonist were withdrawn after week 4 [W4] of 12 weeks of qw DPL [300 mg] or placebo [PBO]. Asthma exacerbation occurrence was the primary endpoint. Fractional exhaled nitric oxide (FeNO) and blood eotaxin-3, TARC and IgE were measured as pharmacodynamic markers. Two AD studies in adults with moderate-to-severe AD evaluated DPL (75, 150, 300 mg) or PBO qw for 4 weeks on itch (5D pruritus questionnaire), Eczema Area and Severity Index (EASI), TARC and total IgE.

## Results

DPL was generally well tolerated in asthma and AD. The most frequent adverse events associated with DPL treatment were nasopharyngitis and headache. Key efficacy endpoints were achieved in both asthma and AD. In asthma, DPL significantly suppressed (mean % change from baseline) TARC (W4: -29 DPL vs. +7 PBO; W12: -26 DPL vs. +8 PBO), eotaxin-3 (W4: -37 DPL vs. +3 PBO; W12: -46 DPL vs. +5 PBO), total IgE (W4: -10 DPL vs. +14 PBO; W12: -37 DPL vs. +6 PBO) and FeNO (mean % change from baseline W4: -40 DPL vs. -5 PBO; W12: -29 DPL vs. +35 PBO). Improvement in FEV1 and the reduction in FeNO were significantly correlated. In AD, 300 mg DPL markedly suppressed TARC (mean -66% DPL vs. -8% PBO) at W4. IgE gradually declined (300 mg, mean -31% DPL vs. +9% for PBO at W12, 9 wks after last dose). TARC levels significantly correlated with the 5D pruritus score at baseline and W4.

## Conclusions

The suppression by DPL of Th2 biomarkers in both asthma and AD concur with improvement in key clinical endpoints. Declines in TARC, as well as eotaxin-3 in asthma, suggest that DPL suppresses Th2-mediated secretion of chemotaxins that perpetuate inflammation. Declines in IgE indicate that DPL suppresses Th2-mediated polarization of Ig-producing cells. A reduction in FeNO demonstrated a reduction in airway inflammation, which correlated with improved lung function (FEV1). The correlation of pruritus with TARC suggests Th2 inflammation may, in part, mediate itch in AD.

